# An Automated Imaging Method for Quantification of Changes to the Endomembrane System in Mammalian Spheroid Models

**DOI:** 10.21769/BioProtoc.5331

**Published:** 2025-06-05

**Authors:** Margaritha M. Mysior, Jeremy C. Simpson

**Affiliations:** Cell Screening Laboratory, School of Biology and Environmental Science, O’Brien Centre for Science, University College Dublin, Dublin, Ireland

**Keywords:** 3D cell culture, Spheroids, Membrane trafficking, Rabs, High-content screening, Fluorescence microscopy

## Abstract

Three-dimensional cell models, such as spheroids, represent a more physiological arrangement in which cells can grow, allowing them to develop cell–cell interactions in all dimensions. The most common methods for growing spheroids are scaffold-based, typically using either extracellular matrix or hydrogels as a physical support for the cellular assembly. One key problem with this approach is that the spheroids that are produced can be highly variable in size and shape. The protocol presented here allows for the systematic production of uniform spheroids in a short time frame by utilising a micropatterned plate. We show that spheroids can be used to investigate fundamental research questions, such as how the endomembrane system is organised in cells. Our protocol can be used in a manual or automated manner, potentially allowing scaling up for screening applications. Furthermore, without the complication of removing the spheroids from the extracellular matrix or hydrogel, as would be required in scaffold-based systems, spheroids can easily be used in other downstream applications.

Key features

• This work builds upon the method developed by Monjaret et al. [1] and establishes a robust method to produce spheroids from HeLa Kyoto cells.

• This protocol generates consistent populations of spheroids that can be used to investigate organelle biology and membrane trafficking pathways.

• Entire spheroids can be analysed in a volumetric manner.

• This method can be used in both manual and automated pipelines, thereby facilitating use in high-throughput and high-content screens.

## Graphical overview


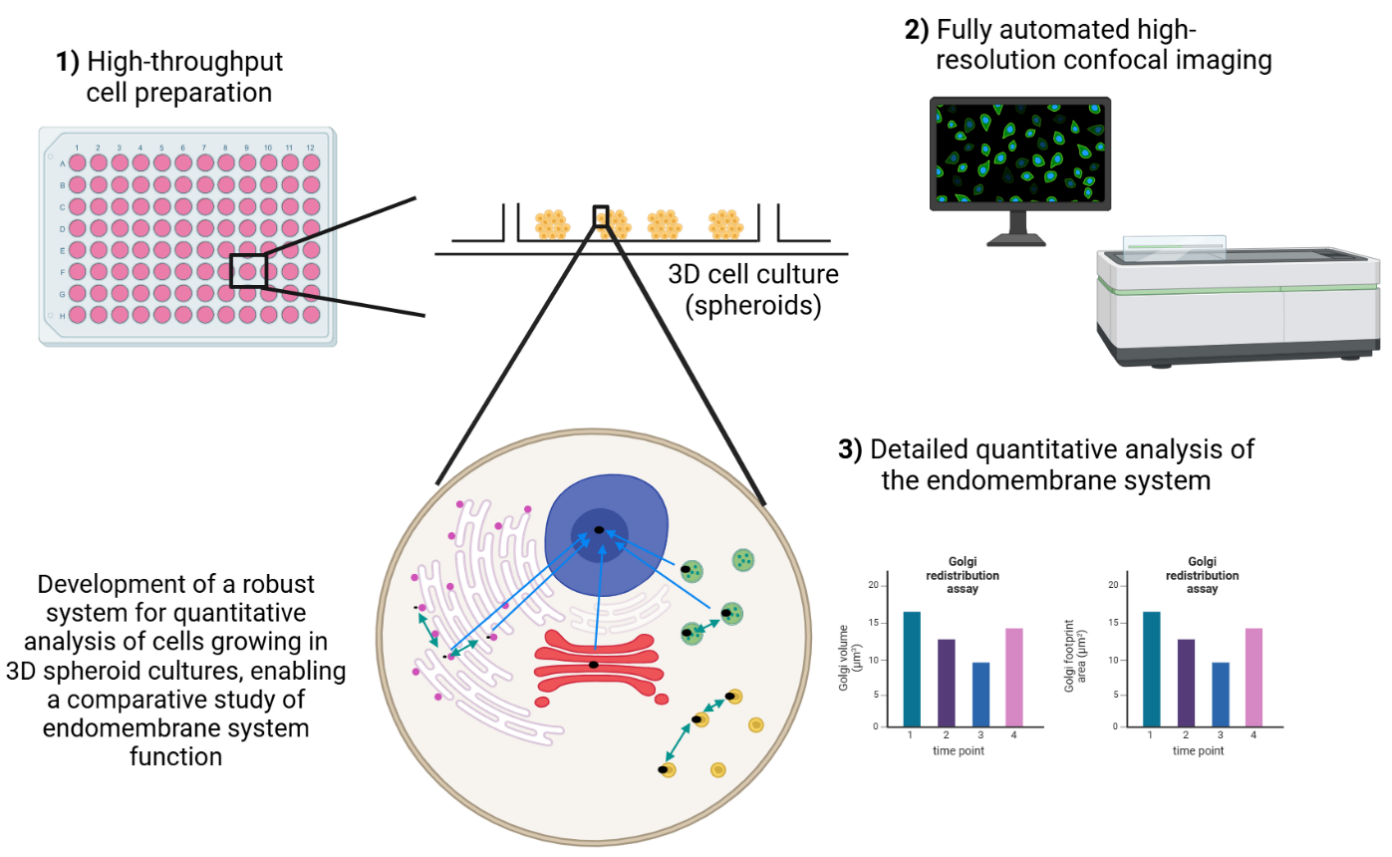


## Background

Three-dimensional (3D) cell models are increasingly utilised in research as they more closely mimic the arrangement of cells in the human body when compared to classical monolayer cultures [2–4]. 3D cell models can be broadly classified into two categories: spheroids and organoids. Organoids are organ-like cell assemblies mainly grown from stem cells [2,4–6]. The primary technique used to grow organoids is an extracellular matrix material with the cells embedded in the matrix, the molecules of which mimic the in vivo environment [7–10]. Spheroids can be grown from established cell lines and stem cells. If cancer cell lines are used, they are termed multicellular tumour spheroids [3,11]. There are many different techniques for growing spheroids. These techniques can be classified into scaffold-based and scaffold-free methods, with each having its particular advantages and disadvantages [12–16]. Many of these methods produce spheroids that are heterogeneous in both size and shape, making comparisons across any population challenging. Scaffold-free methods most commonly utilise round-bottom ultra-low attachment plates for spheroid growth, which, although allowing high uniformity of spheroid size, suffer from the key disadvantage of only producing a single spheroid per well. This therefore precludes their use in large-scale screening studies. The most suitable method for growing spheroids also depends on the cell lines and types needed, in addition to the specific research question being addressed.

3D cell models have a wide number of applications, including in cancer research, developmental cell biology, toxicology studies, and drug development [12,14,15,17–19]. However, to date, fundamental research areas have largely underutilised them [16] despite their potential being proposed almost twenty years ago [20,21]. The protocol that we have developed allows the routine production and use of uniform-sized spheroids in fundamental and applied research studies. This has the potential to significantly simplify the use of spheroids in many important applications, such as toxicity studies. These experiments are often carried out by growing spheroids in basement membrane extracts, which, although having the advantage of being relatively technically simple, results in an extremely diverse population of spheroids being produced within a single well [18]. Importantly, the size of spheroids produced allows the investigation of cellular behaviour at a subcellular scale, and the entire spheroid can be fully imaged with confocal microscopy, allowing them to be used for volumetric image analysis measurements. Together, this means that information can be extracted at subcellular, cellular, and whole-spheroid levels from the same sample. Furthermore, our method produces several hundred spheroids per well in a multi-well plate, thereby enabling their easy use in downstream applications, such as RNA extraction and qPCR.

## Materials and reagents

**Biological materials**

1. HeLa Kyoto cells (human cervical cancer cell line, RRID; CVCL-1922)

2. HeLa Kyoto cells stably expressing EGFP (generated in-house [22])

3. HeLa Kyoto cells stably expressing EGFP-Rab5A (generated in-house [22])

4. HeLa Kyoto cells stably expressing EGFP-Rab6A (generated in-house [22])

**Reagents**

1. Dulbecco’s modified Eagle medium (DMEM) with L-Glutamine, pyruvate, 1 g/L glucose (Life Technologies Europe BV, Gibco, catalog number: 31885023); store at 4 °C

2. Value heat-inactivated (HI) fetal bovine serum (FBS) (Life Technologies Europe BV, Gibco, catalog number: A5256801); store at -20 °C

3. L-Glutamine solution (200 mM) (Life Technologies Europe BV, Gibco, catalog number: 25030024); store at -20 °C

4. 0.05% Trypsin-EDTA (1×), phenol red (Life Technologies Europe BV, Gibco, catalog number: 25300054); store at -20 °C

5. G418 sulfate (50 mg/mL) (Fisher Scientific, Corning, catalog number: 30-234-CI); store at 4 °C

6. PBS tablets (Fisher Scientific, catalog number: 12821680); store at room temperature (RT)

7. Dimethyl sulfoxide (DMSO) (VWR, catalog number: A3672.0100); store at RT

8. Glycine (Fisher BioReagents, catalog number: BP3811); store at RT

9. Bisbenzimide H 33342 trihydrochloride (Hoechst 33342) (Merck, catalog number: 14533); store at 4 °C

10. Triton X-100 (Merck, catalog number: T9284-500ml); store at RT

11. Rabbit anti-GM130 (clone: D6B1) (Cell Signalling Technologies, catalog number: 12480); store at -20 °C

12. Mouse anti-EEA1 (clone: 14/EEA1) (BD Biosciences, catalog number: 610457); store at -20 °C

13. Mouse anti-LAMP1 (clone: H4A3) (Developmental Studies Hybridoma Bank, catalog number: H4A3, supernatant); store at 4 °C

14. Alexa Fluor 568 goat anti-rabbit IgG (H+L) highly cross-adsorbed (Thermo Fisher, catalog number: A11036); store at -20 °C

15. Alexa Fluor 647 goat anti-mouse IgG (H+L) highly cross-adsorbed (Thermo Fisher, catalog number: A21236); store at -20 °C

16. Sodium azide (Merck, catalog number: S2002-25g); store in a well-ventilated place

**Caution:** Sodium azide is toxic.

17. Brefeldin A (BFA) (Merck, catalog number: B7651); store at 4 °C

18. Nocodazole (Merck, catalog number: M1404); store at 4 °C

19. Deionised water (dH_2_O)

20. Paraformaldehyde (PFA) (Merck, catalog number: P6148), store at 4 °C

**Caution:** PFA is toxic.

21. Calcium chloride dihydrate (Merck, catalog number: C3306)

22. Magnesium chloride hexahydrate (Fisher Scientific, catalog number: M/0600/53)

**Solutions**

1. Complete medium (see Recipes)

2. Complete medium with G418 (see Recipes)

3. 1× PBS (see Recipes)

4. Hoechst 33342 solution (see Recipes)

5. 1 M glycine solution (see Recipes)

6. 30 mM glycine solution (see Recipes)

7. 10% Triton X-100 solution (see Recipes)

8. 0.5% Triton X-100 solution (see Recipes)

9. 10% sodium azide solution (see Recipes)

10. BFA solution (see Recipes)

11. Nocodazole solution (see Recipes)

12. 1 M calcium chloride (see Recipes)

13. 1 M magnesium chloride (see Recipes)

14. 3% PFA (see Recipes)

**Recipes**

**1. Complete medium**

Thaw the HI FBS and L-Glutamine at 37 °C for 1 h or at 4 °C overnight. Remove 55 mL from a fresh bottle of DMEM and put it into two 50 mL conical tubes. Mix HI FBS and L-Glutamine with DMEM. Store at 4 °C. All steps are carried out in a Class II biological safety cabinet.

**Table:** 

Reagent	Final concentration	Quantity or volume
DMEM 1 g/L glucose		445 mL
HI FBS	10%	50 mL
L-Glutamine	1%	5 mL
Total		500 mL

**2. Complete medium with G418**

Follow the same steps as in Recipe 1; remove 62 mL from a fresh bottle of DMEM. Additionally, add the G418. Store at 4 °C. All steps are carried out in a Class II biological safety cabinet.

**Table:** 

Reagent	Final concentration	Quantity or volume
DMEM 1 g/L glucose		438 mL
HI FBS	10%	50 mL
L-Glutamine	1%	5 mL
G418	700 μg/mL	7 mL
Total		500 mL

**3. 1× PBS**

Dissolve one tablet in 100 mL of dH_2_O. Prepare one bottle for cell culture and one bottle for main lab work. The 1× PBS that is used in cell culture should be autoclaved; 1× PBS for main lab work does not necessarily need to be autoclaved. Store at RT.

**Table:** 

Reagent	Final concentration	Quantity or volume
PBS tablet	1×	1 tablet
dH_2_O	n/a	100 mL
Total		100 mL

**4. Hoechst 33342 solution**

Weigh 1 mg of Hoechst 33342 and put it into a tube with 1 mL of dH_2_O. Vortex the tube. Once the Hoechst 33342 has dissolved, centrifuge at 140× *g* for 1 min. Prepare 50 μL aliquots and store at -20 °C. These are stable for at least one year. Protect the aliquots from light when in use.

**Table:** 

Reagent	Final concentration	Quantity or volume
Hoechst 33342	1 mg/mL	1 mg
dH_2_O	n/a	1 mL
Total		1 mL

**5. 1 M glycine solution**

Weigh the glycine and put it into a 50 mL conical tube. Add the PBS (not necessarily autoclaved) and vortex the conical tube to dissolve the glycine. Store at 4 °C.

**Table:** 

Reagent	Final concentration	Quantity or volume
Glycine	1 M	3.753 g
PBS (Recipe 3)	n/a	50 mL
Total		50 mL

**6. 30 mM glycine solution**

Add 1.5 mL of 1 M glycine into 48.5 mL of PBS (not necessarily autoclaved) in a 50 mL conical tube. Vortex the conical tube to create a homogenous solution. Store at 4 °C.

**Table:** 

Reagent	Final concentration	Quantity or volume
1 M glycine (Recipe 5)	30 mM	1.5 mL
PBS (Recipe 3)	n/a	48.5 mL
Total		50 mL

**7. 10% Triton X-100 solution**

Slowly aspirate the Triton X-100 and add it to the PBS (not necessarily autoclaved). Vortex the conical tube to create a homogenous solution. Store at 4 °C.

**Table:** 

Reagent	Final concentration	Quantity or volume
Triton X-100	10%	5 mL
PBS (Recipe 3)	n/a	45 mL
Total		50 mL

**8. 0.5% Triton X-100 solution**

Add the appropriate amount of 10% Triton X-100 solution to PBS (not necessarily autoclaved). Vortex the conical tube to create a homogenous solution. Store at 4 °C.

**Table:** 

Reagent	Final concentration	Quantity or volume
10% Triton X-100	0.5%	2.5 mL
PBS (Recipe 3)	n/a	47.5 mL
Total		50 mL

**9. 10% sodium azide solution**

Wear a protective mask and gloves when weighing the powder in a fume hood. Vortex the conical tube with sodium azide and dH_2_O to dissolve the sodium azide. **Caution:** Sodium azide is toxic.

**Table:** 

Reagent	Final concentration	Quantity or volume
Sodium azide	10% (w/v)	1 g
dH_2_O	n/a	10 mL
Total		10 mL

**10. BFA solution**

Add the DMSO directly into the vessel containing the BFA powder. Vortex the vessel to dissolve the BFA in DMSO. Prepare 50 μL aliquots and store at -20 °C. All steps are carried out in a Class II biological safety cabinet.

**Table:** 

Reagent	Final concentration	Quantity or volume
BFA	10 mg/mL	5 mg
DMSO	n/a	500 μL
Total		500 μL

**11. Nocodazole solution**

Add the DMSO directly into the vessel containing the nocodazole powder. Vortex the vessel to dissolve the nocodazole in DMSO. Prepare 50 μL aliquots and store at -20 °C. All steps are carried out in a Class II biological safety cabinet.

**Table:** 

Reagent	Final concentration	Quantity or volume
Nocodazole	10 mM	2 mg
DMSO	n/a	660 μL
Total		660 μL

**12. 1 M calcium chloride solution**

Weigh the calcium chloride and put it into a 50 mL conical tube. Add the PBS (not necessarily autoclaved) and vortex the conical tube to dissolve the calcium chloride. Store at RT.

**Table:** 

Reagent	Final concentration	Quantity or volume
Calcium chloride	1 M	1.47 g
PBS	n/a	10 mL
Total		10 mL

**13. 1 M magnesium chloride**

Weigh the magnesium chloride and put it into a 50 mL conical tube. Add the PBS (not necessarily autoclaved) and vortex the conical tube to dissolve the magnesium chloride. Store at RT.

**Table:** 

Reagent	Final concentration	Quantity or volume
Magnesium chloride	1 M	2.03 g
PBS	n/a	10 mL
Total		10 mL

**14. 3% PFA**

Heat 400 mL of the autoclaved PBS to 60–70 °C in a fume hood while stirring on a hot-plate stirrer. Turn off the fume hood and weigh the PFA. Wear a protective mask and gloves when weighing the powder in a fume hood. Add the PFA powder carefully into the PBS. Clean everything with a damp paper before turning the fume hood back on. Keep the solution heated between 60 and 70 °C. Wait until the PFA has fully dissolved (**Tip:** Adding a few droplets of 5 M sodium hydroxide helps to dissolve the PFA faster). After the PFA has dissolved, add 1 M calcium chloride and 1 M magnesium chloride. Adjust the PFA solution to the final volume with the remaining PBS. Adjust the pH to 7.4 with either sodium hydroxide or hydrochloric acid. Filter the PFA solution through a Stericup vacuum filter. Prepare aliquots of the PFA solution and store them at -20 °C. These are stable for at least one year.

**Table:** 

Reagent	Final concentration	Quantity or volume
PFA	3%	15 g
1 M calcium chloride (Recipe 12)	0.1 mM	50 μL
1 M magnesium chloride (Recipe 13)	0.1 mM	50 μL
PBS	n/a	500 mL
Total		500 mL

**Laboratory supplies**

1. Tissue culture dish (Nunclon) with lid, polystyrene, radiation, 92 mm × 17 mm (Life Technologies Europe BV, catalog number: 150350)

2. 5 mL sterile serological pipette (Fisher Scientific, catalog number: 11829660)

3. 10 mL sterile serological pipette (Fisher Scientific, catalog number: 11839660)

4. 25 mL sterile serological pipette (Fisher Scientific, catalog number: 11517752)

5. 50 mL conical tubes (Corning, catalog number: 430828)

6. 2 mL microcentrifuge tube (Eppendorf, catalog number: 0030120086)

7. 1.5 mL microcentrifuge tube (Eppendorf, catalog number: 0030120094)

8. 1,000 μL tips (Eppendorf, catalog number: 0030000927)

9. 200 μL tips (Gilson, catalog number: F161930)

10. 10 μL tips (Gilson, catalog number: F161631)

11. 125 μL sterile GRIPTIPS, 5 XYZ racks of 384 tips (Integra Biosciences, catalog number: 6464)

12. 125 μL non-sterile GRIPTIPS, 5 XYZ racks of 384 tips (Integra Biosciences, catalog number: 6463)

13. 10 mL disposable reservoirs, sterile, SureFlo^TM^ anti-sealing array (Integra Biosciences, catalog number: 4373)

14. 300 mL robotic reservoir, flat bottom, sterile (Thermo Scientific, Nunc, catalog number: 10723363)

15. Scepter 2.0 handheld automated cell counter (Merck, catalog number: PHCC20060)

16. Scepter 60 μm sensors (Merck, catalog number: PHCC60050)

17. Pasteur pipette, glass unplugged, 230 mm (Fisher Scientific, catalog number: 1156-6963)

18. Stericup quick release vacuum filtration system (Merck, catalog number: S2HVU05RE)

19. CYTOO plates 96RW custom fibronectin-coated, DC45/P300 (CYTOO, catalog number: 20-950-10); store at 4 °C

## Equipment

1. Class II biological safety cabinet (Faster)

2. CO_2_ incubator (New Brunswick Scientific, model: Innova CO-170)

3. Vacusafe vacuum aspirator with Vacuboy (Integra Biosciences, catalog numbers: 158300 and 155500)

4. Pipetboy Pro (Integra Biosciences)

5. Research Plus variable adjustable volume pipettes, volume: 100–1,000 μL (Eppendorf, catalog number: 3123000063)

6. Research Plus variable adjustable volume pipettes, volume: 20–200 μL (Eppendorf, catalog number: 3123000055)

7. Research Plus variable adjustable volume pipettes, volume: 2–20 μL (Eppendorf, catalog number: 3123000039)

8. Research Plus variable adjustable volume pipettes, volume: 0.5–10 μL (Eppendorf, catalog number: 3123000020)

9. Waterbath (Grant, model: JB nova)

10. Light microscope (Olympus, model: CKX53); objectives: 10× and 20×

11. Viaflo 96 (with 125 μL head and 2 spring-loaded plate holders) (Integra Biosciences, catalog number: 6001)

12. Viaflo electronic pipette 8-channel, 5–125 μL (Integra Biosciences, catalog number: 4722)

13. Laboratory centrifuge with rotor adaptor for 15 and 50 mL conical tubes (Eppendorf, model: 5810 R, catalog number: 5811000065)

14. Vortex (Merck, catalog number: Z258423)

15. Mini microfuge (VWR)

16. Tube racks

17. Hot-plate stirrer

18. Opera Phenix high-content screening system (Revvity)

## Software and datasets

1. Harmony (Revvity, Version 4.8 and 4.9); requires a license

2. All image data relevant to this paper are available from the BioImage Archive (https://www.ebi.ac.uk/biostudies/bioimages/studies) at DOI:10.6019/S-BIAD1259

## Procedure

In this protocol, we grow spheroids using CYTOO plates. The particular CYTOO plate used here has a disc micropattern diameter of 45 μm and a pitch between each disc micropattern of 300 μm. Such plates can be custom-designed with various micropattern diameters and pitch sizes depending on the cells grown and the application. The disc micropatterns are coated with fibronectin to improve cell adherence.

**A. Preparation of plates**

1. Take out the CYTOO plate from the fridge and warm it up at RT in the Class II biological safety cabinet for 1 h.

*Note: While the plate is warming up, you can prepare the cells for plating.*

2. After 1 h, add 100 μL of complete medium (see Recipes) into the well.

3. Incubate the plate in the humidified incubator at 37 °C with 5% CO_2_ for at least 10 min.

*Note: After this incubation, the plate is ready for cell plating.*

**B. Preparation of cells for plating**

In this protocol, HeLa Kyoto cells are used to grow spheroids on CYTOO plates. However, the following protocol can be applied to any cell line that can be grown as spheroids using other methods. For a different cell line, the adhesion molecule, number of cells seeded, and number of growth days need to be optimised. All the media and trypsin used for the cell culture should be warmed up to 37 °C in the water bath prior to use.

1. When the cells reach 80% confluency in a 10 cm culture dish, approximating 4 million cells (in 12 mL complete medium), wash them with 2 mL of 1× PBS. Using the Vacuboy, aspirate the PBS off and add 3 mL of fresh 0.05% trypsin-EDTA. Put the cells into the humidified incubator at 37 °C with 5% CO_2_ and incubate for 3–4 min.

2. When the cells are detached, add 7 mL of complete medium. Wash the cell culture dish to fully detach the cells. Transfer the 10 mL of cell suspension into a 50 mL conical tube. Pipette the cell suspension up and down to produce a homogenous suspension.

*Note: For this protocol, HeLa Kyoto (parental) and HeLa Kyoto cells stably expressing EGFP, EGFP-Rab5A, or EGFP-Rab6A were used. Parental HeLa Kyoto cells were grown in complete medium (see Recipe 1), and HeLa Kyoto cells stably expressing EGFP, EGFP-Rab5A, or EGFP-Rab6A were grown in complete medium containing 700 μg/mL G418 (see Recipe 2).*

3. Pipette 1 mL of cell suspension into a 1.5 mL microcentrifuge tube for cell counting.

4. Turn on the Scepter cell counter and insert the 60 μm sensor into it. Then, insert the sensor into the 1.5 mL microcentrifuge tube containing the cell suspension and count the number of cells.

*Note: A hemocytometer counting chamber or other automated cell counting equipment can also be used to count the number of cells.*

5. Calculate the total number of cells in your cell suspension by multiplying the number of cells counted by the Scepter times 10.

6. Centrifuge the cells at 135× *g* for 4 min at RT.

7. Carefully aspirate the supernatant with the Vacuboy and add the appropriate amount of complete medium to the cell pellet to create a cell suspension of 1 × 10^6^ cells/mL. Resuspend the cell pellet in complete medium.

8. Preparation of cell suspension: plate 5,000 cells per well in 100 μL of complete medium into each well of the CYTOO plate. For this, 5 μL of the 1 × 10^6^ cells/mL cell suspension is mixed with 95 μL of complete medium. Prepare sufficient cell suspension for each cell line for plating (either in a microcentrifuge tube or a conical tube).

*Note: To prepare sufficient cell suspension for plating, the following formula was used: number of wells + 5 wells. If you are using automated cell plating equipment (e.g., a Multidrop), you should add 10% extra wells.*

**Pause point:** Cells can be kept in the tube in the Class II biological safety cabinet until the plate is ready to use.

9. Plating of cells into the plate: Carefully pipette the cell suspension created in step B8 up and down to create a homogenous cell suspension. Pipette 100 μL of cell suspension into each well containing 100 μL of complete medium (see section A) by placing the pipette tip over the middle of the well and carefully releasing the cell suspension.

*Note: For large-scale spheroid production, this step can be carried out with automated cell plating equipment (e.g., a Multidrop).*

10. Place the plate into a humidified incubator at 37 °C with 5% CO_2_. Let the cells grow into spheroids for 3 days. Monitor the spheroid growth with a light microscope over the 3 days.

*Note: Initial cell adhesion to the micropattern can be checked after 1 h. Non-attached cells can be gently washed off with a medium exchange the following day by removing 150 μL of complete medium and replacing it with 150 μL of fresh complete medium. Repeat the wash 2–3 times. The number of growth days depends on the cell line used and the desired size of the spheroids. For longer periods of growth, it is recommended to exchange the medium every two days, as described for the well wash above.*

**C. Immunostaining of spheroids**

For this part of the protocol, it is important to be gentle with the spheroids. Always use an electronic 8-channel pipette to aspirate and dispense liquids from/to the well at the slowest pipetting speed. The plate is always covered with aluminium foil for the incubation periods in order to minimise exposure of the fluorophores to light. If all wells in the plates are used, these steps can be carried out with pipetting robots (e.g., Viaflo 96). The PBS used to produce the primary and secondary antibody solutions, as well as for the washes, does not necessarily need to be autoclaved.

1. Remove the media from the wells.

2. Add 100 μL of 3% PFA (see Recipe 14) and incubate for 1 h at RT.

3. Quench with 100 μL of 30 mM glycine in PBS for 30 min.

4. Wash the spheroids twice with PBS.

**Pause point:** You can pause the spheroid staining here and continue within a few days. Store the plate at 4 °C.

5. Permeabilise the spheroids with 100 μL of 0.5% Triton X-100 for 1 h at RT.

6. Wash the spheroids twice with PBS.

7. Incubate the spheroids with 100 μL of primary antibody solution in PBS for 2 h at RT. For this, prepare a fresh primary antibody solution by pipetting the primary antibody and 0.02% sodium azide in the appropriate volume of PBS. The rabbit anti-GM130 antibody was used at a dilution of 1:500, and the mouse anti-EEA1 and mouse anti-LAMP1 antibodies were used at 1:200.

*Note: The dilution depends on the specific antibody used. The dilution of the anti-LAMP1 antibody may vary depending on the supernatant batch. Test new batches before usage. Use the stock of sodium azide (see Recipe 9) to produce a final concentration of 0.02% sodium azide. Alternatively, antibody incubation can be carried out overnight at RT or 4 °C.*

8. Wash the spheroids twice with PBS.

9. Incubate the spheroids with 100 μL of secondary antibody solution in PBS for 2 h at RT. For this, prepare a fresh solution by pipetting the secondary antibody, 0.2 μg/mL Hoechst 33342, and 0.02% sodium azide in the appropriate volume of PBS. The secondary antibodies goat anti-rabbit Alexa Fluor 568 and goat anti-mouse Alexa Fluor 647 were used at a dilution of 1:400.

*Note: The antibody dilution depends on the specific antibody used. Highly cross-adsorbed secondary antibodies that show high photostability and high brightness are recommended. Use the Hoechst 33342 stock (see Recipe 4) and sodium azide (Recipe 9) to produce a final concentration of 0.2 μg/mL Hoechst 33342 and 0.02% sodium azide, respectively. Alternatively, antibody incubation can be carried out overnight at RT or 4 °C.*

10. Wash the spheroids twice with PBS.

11. Store the plates at 4 °C covered in aluminium foil.

*Note: Image the spheroids within 2 weeks to achieve a good fluorescent signal strength.*

**D. Drug inhibitor assay in spheroids**

Two common inhibitors of membrane trafficking events can be used to show the applicability of spheroids in studying membrane trafficking events. Again, all tests should be carried out using an electronic 8-channel pipette to aspirate and dispense liquids from/to the well at the slowest pipetting speed. For plates in which all wells have been used, these steps can be carried out with pipetting robots (e.g., Viaflo 96).

1. First, freshly prepare the BFA (final concentration 10 μg/mL) and nocodazole (final concentration 7.5 μM) solutions in prewarmed complete medium.

*Note: To prepare the solutions, use the stock solutions of BFA (see Recipe 10) and nocodazole (see Recipe 11).*

2. Remove the medium from the spheroid-containing wells/plate.

3. Add 100 μL of drug solution (either BFA or nocodazole).

4. Incubate the spheroids for 30 min at 37 °C in the humidified incubator with 5% CO_2_.

5. Continue with the fixation and immunofluorescence protocol as described in section C.

## Data analysis

**Image acquisition and analysis of spheroids**

Imaging was performed with an Opera Phenix high-content screening (HCS) system. Other HCS systems can be used, but they should have the following features: spinning disk confocal, pre-scan and re-scan capabilities, and water-immersion objectives.

**Image acquisition**

**Figure 1.**
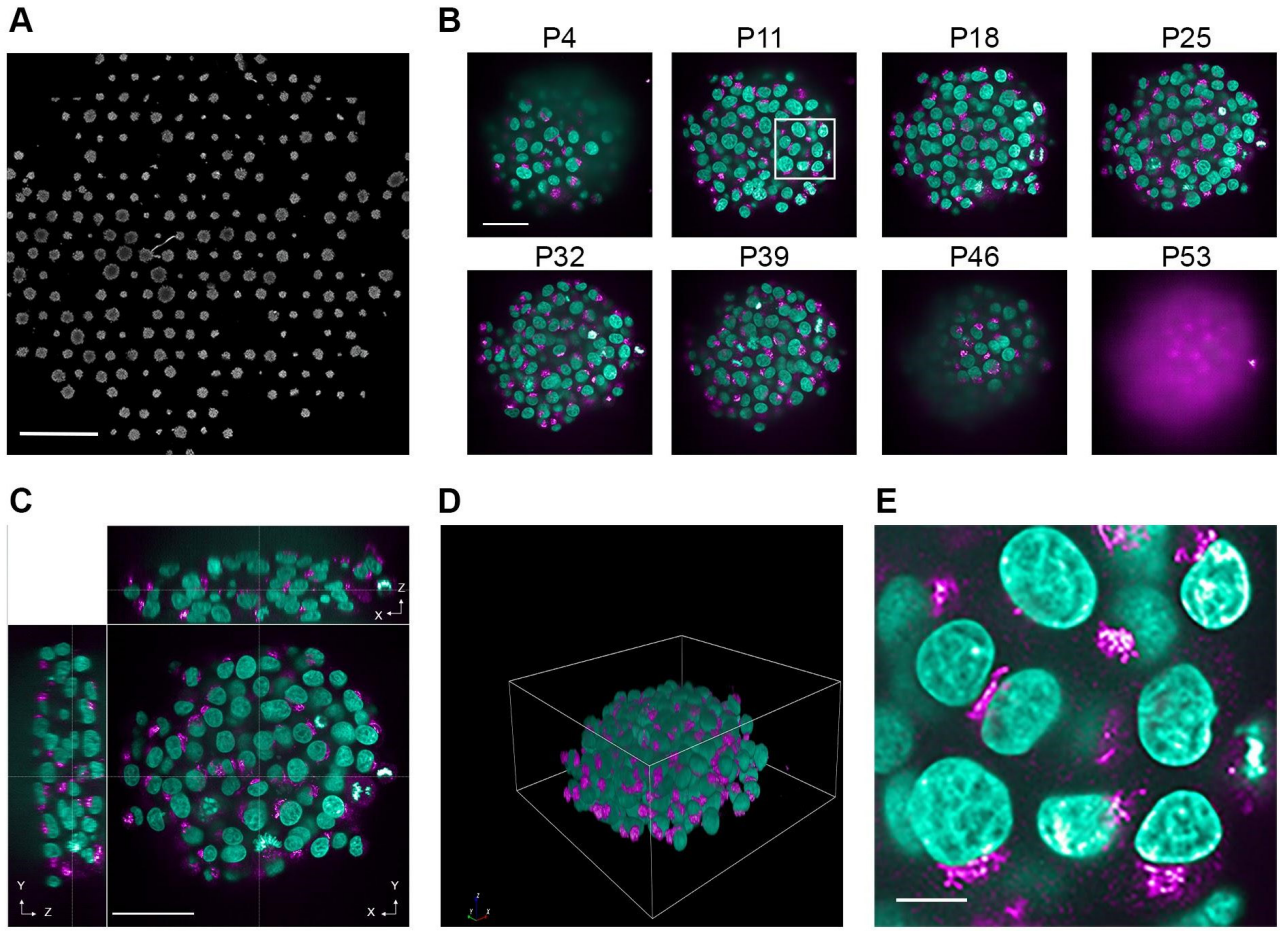
Various levels of information from spheroid image data. Representative images showing different levels of information that can be acquired from spheroids. Spheroids were grown for three days on a CYTOO DC45-P300FN plate, fixed, and stained with Hoechst 33342 (nuclei, green); anti-GM130 antibodies were used to immunostain the Golgi apparatus (magenta). Images were acquired with an Opera Phenix high-content screening microscope with either the 20×/1.0 NA water objective (A) or 63×/1.15 NA water objective (B, C, and E). (A) Overview of one well. Scale bar: 1 mm. (B) Individual images from various z-planes through a spheroid. This panel shows eight example planes (P) from various positions across the depth of the spheroid. The white box represents the area that was used for the image zoom in panel E. Scale bar: 50 μm. (C) XYZ views of a spheroid. Scale bar: 50 μm. (D) Rendered 3D view of a spheroid. (E) Zoom image of individual cells grown in a spheroid. Scale bar: 10 μm.

To reduce the imaging time and data size, each well of the plate should be imaged with a low-magnification objective. This *pre-scanning* is best performed with a 20×/1.0 NA water immersion objective (Figure 1A) to allow identification of the spheroid population in the well. Typically, 10 spheroids per well are then selected to be fully imaged with a 63×/1.15 NA water immersion objective.

1. Insert the plate into a high-content imaging system.

*Note: The plate should be at RT to avoid focus drift during image acquisition.*

2. Prepare the pre-scan experiment file by defining the following parameters:

a. Select the appropriate lasers, laser power, and exposure time to excite the Hoechst 33342 stain.

b. Select the confocal plane where most spheroids are in focus.

c. Choose the position for the 7 × 7 tile that should be pre-scanned.

d. Add your pre-scan pipeline to identify the spheroids based on the Hoechst 33342 signal.

3. Prepare the main imaging experiment file by defining the following parameters:

a. Select the appropriate lasers, laser power, and exposure times to excite the fluorophores.

b. Choose sequential acquisition of the channels to avoid crosstalk.

c. Select the z-stack range and interval between the different individual z-planes (Figure 1B).

4. Before you start imaging, flush the water objective.

*Note: Usual exposure times were between 120 and 500 ms at 100% laser power; typically, 65 planes, each 1 μm apart, were acquired for the rescan. The following excitation and emission wavelengths were used for the various fluorescent dyes and secondary antibodies: Hoechst 33342, ex. 405 nm, em. 435–550 nm; EGFP, ex. 488 nm, em. 500–550 nm; Alexa Fluor 568, ex. 561 nm, em. 570–630 nm; Alexa Fluor 647, ex. 640 nm, em. 650–760 nm.*

**Image analysis**

A volumetric analysis of the full spheroids (Figure 1C, D), as well as individual cells of the spheroid (Figure 1E), is carried out. In order to do this, the Harmony image analysis software is utilised.

1. First, segment the spheroids using the channel of choice. Here, spheroids are segmented with the Alexa 647 channel.

2. Next, segment the nuclei using the Hoechst 33342 channel.

3. Segment the cytoplasm of the cells using the residual Hoechst 33342 signal in the cell. For cells expressing the various EGFP-tagged proteins, the EGFP signal intensity is measured, and cells deemed not to express EGFP are discarded from the analysis.

*Note: If you are using a stain for the cell membrane or cytoplasm, this stain can be used to segment the cell cytoplasm instead.*

4. Filter the channel for the organelle markers to enhance the signal-to-noise ratio for easier organelle segmentation. Typically, for large, distinct organelles such as the Golgi apparatus, a Gaussian filter in combination with a *calculate image* step is applied, whereas for smaller organelles such as early endosomes or lysosomes, a *texture filter* is applied. The *calculate image* step creates a new image, which can be used to segment the object of interest. We typically divide the Gaussian filtered image by the original image to create an image with clearer boundaries of the object of interest. The *texture filter* step uses texture features (here, the spot feature) to again create clearer boundaries of the object of interest for segmentation.

5. After filtering, segment the individual organelles within the cells.

6. Measurements can be applied to the spheroid, cell, or organelle population. Typically, features measured are volume, surface area, number of objects, intensity, and texture. Please find the exact details of the image analysis pipeline in the supplementary material of [22].

## Validation of protocol

This protocol has been used and validated in the following research article:

• Mysior and Simpson [22]. An automated high-content screening and assay platform for the analysis of spheroids at subcellular resolution. *PLoS One*, 19(11), e0311963. https://doi.org/10.1371/journal.pone.0311963

In the supplementary information in this article, readers will also find seven supplementary tables detailing, step-by-step, seven different image analysis pipelines. These use the commercial imaging analysis software Harmony that is provided with Opera Phenix microscope.

In general, approximately 90% of micropatterns were occupied by cells shortly after seeding. After 3 days of growth, approximately 60%–80% of the micropatterns contained spheroids [22]. At this point, using a pre-scan function on the microscope, spheroids with particular size characteristics can be selected for higher resolution imaging. Using this approach, primary characterisation of the spheroids showed that each had a similar average area, and that this was also consistent between the different cell lines (see Figure 1C in [22]). Furthermore, the average spheroid volume (Figure 2B in [22]) and the number of nuclei per spheroid (Figure 2D in [22]) were in the same range for all cell lines. The overexpression of some proteins, particularly those that play a role in cell division or assembly of the plasma membrane, can result in changes to spheroid size and other properties. For example, we found that spheroids overexpressing EGFP-Rab6A were on average 20% smaller than the other spheroids (Figure 2A, D in [22]). However, a key advantage of this method is that several hundred spheroids can be generated per well, compared to more commonly used methods such as U-bottomed ULA plates and hanging drop methods, which typically produce only one spheroid per well or drop. Other methods that produce large numbers of spheroids per well, for example, through the use of extracellular matrix, result in a much higher heterogeneity of spheroid size, with typical size differences being more than 10-fold in a single fold [18,23].

The small, but relatively uniform size of the spheroids produced in the current protocol allowed for the acquisition of volumetric information from various organelles of the endomembrane system (see Figures 3, S2, and S2 in [22]). Quantitative subcellular information from the spheroids was extracted from the Golgi apparatus, early endosomes, and lysosomes. Various parameters, such as the volume of the organelle, its surface area, and the number of fragments or distinct organelles, can be extracted from the images. Quantitative analysis of the Golgi volume in cells grown as spheroids measured approximately 125 μm^3^ (see Figure 3E in [22]). The total volume occupied by early endosomes and lysosomes in the cell was 120 and 30 μm^3^, respectively (see Figures S2E and S3E in [22]). These examples demonstrate the potentially rich quantitative information that can be extracted from individual cells in spheroids. This approach could, in principle, be easily adapted for most other subcellular structures.

Furthermore, in the work on which this protocol is based, we were also able to conduct widely used functional assays in the field of membrane trafficking using the spheroid model, namely BFA and nocodazole treatment, to provide proof-of-principle data. A reduction in Golgi volume, together with an increase in the number of Golgi fragments per cell after BFA and nocodazole treatment, respectively, could be measured (see Figures 4C, D and S4C, D in [22]). Alongside changes to organelles, we were able to quantify changes in the EGFP-Rab5A and EGFP-Rab6A patterns in cells grown as spheroids after BFA and nocodazole-induced treatments, respectively, utilising, for example, texture features (see Figures 5 and S5 in [22]). Again, this demonstrates the potentially wide applicability of the protocol detailed here.

## General notes and troubleshooting

**General notes**

1. If optimisation steps are carried out at the beginning, and only a few wells of the plate have been used for this, the remaining wells can be preserved by adding some PBS and sealing them with parafilm. After using the plate, it should be stored at 4 °C. In our experience, it should be used within four weeks of being removed from its original packaging.

2. Different cell lines can be used to grow spheroids on these plates (see [1]).

3. The micropattern disc size of 45 μm as used here is the best for growing spheroids from HeLa Kyoto cells. A larger disc size is not suitable for HeLa Kyoto cells as they no longer form spheroids.

4. The growth time is dependent on the desired size of the spheroids. Longer incubation times can be used to grow larger spheroids or when using slow-growing cells.

**Troubleshooting**

Problem 1: Cells do not adhere to the micropattern.

Possible cause: The wrong adhesive molecule was used.

Solution: Try a variety of adhesive molecules, such as fibronectin, collagen, and poly-lysine, for each cell line.

Problem 2: Spheroids are seen to fuse.

Possible cause: Too many cells plated, or the distance between the micropatterns is too small.

Solution: Plate fewer cells at the beginning and wash the wells more frequently, or acquire a plate on which the micropatterns have a larger pitch between each other.

Problem 3: No spheroids are left after medium exchange or processing for immunofluorescence.

Possible cause: Harsh liquid exchange or spheroids are not adherent.

Solution: Exchange any liquids as slowly as possible.

Problem 4: Cells do not form spheroids.

Possible cause: Cells do not tend to form spheroids if the growth time is too short or the micropattern size is too large.

Solution: Try a cell line that naturally grows in clumps, increase the growth time, or use a smaller micropattern size.

Problem 5: The staining did not work.

Possible cause: Issues with the antibodies/dyes.

Solution: If using new antibodies/dyes, try them first on cultured monolayer cells before using them to stain spheroids. If established antibodies/dyes are used, increase the incubation time and/or the concentration of the antibody/dye.
